# Rust Formation Mechanism on Low Alloy Steels after Exposure Test in High Cl^−^ and High SO*_x_* Environment

**DOI:** 10.3390/ma10020199

**Published:** 2017-02-17

**Authors:** Toshiyasu Nishimura

**Affiliations:** Corrosion Resistant Steel Group, National Institute for Materials Science (NIMS), Tsukuba, Ibaraki 305-0047, Japan; NISHIMURA.Toshiyasu@nims.go.jp; Tel.: +81-29-859-2127

**Keywords:** atmospheric corrosion, low alloy steel, rust, Cl^−^, SO*_x_*, nickel, chromium, transmission electron microscopy, Raman spectroscopy

## Abstract

Exposure tests were performed on low alloy steels in high Cl^−^ and high SO*_x_* environment, and the structure of the rust were analyzed by TEM (Transmission Electron Microscopy) and Raman Spectroscopy. In the exposure test site, the concentrations of Cl^−^ and SO*_x_* were found to be high, which caused the corrosion of the steels. The conventional weathering steel (SMA: 0.6% Cr-0.4% Cu-Fe) showed higher corrosion resistance as compared to the carbon steel (SM), and Ni bearing steel exhibited the highest one. Raman spectroscopy showed that the inner rust of Ni bearing steel was mainly composed of α-FeOOH and spinel oxides. On the other hand, SMA contained β- and γ-FeOOH in inner rust, which increased the corrosion. TEM showed that nano-scale complex iron oxides containing Ni or Cr were formed in the rust on the low alloy steels, which suppressed the corrosion of steels in high Cl^−^ and high SO*_x_* environment.

## 1. Introduction

As the economy of East Asia grows rapidly, the corrosion of infrastructure is becoming a serious problem. The corrosion by airborne salt particles in coastal areas is reported as severer, which is caused by Cl^−^ ion from the sea. However, although the corrosion in high SO*_x_* environment is thought to be heavy, there is little information on this case. Besides, there is no information on the corrosion behavior of steels under high Cl^−^ and high SO*_x_* environment. As there are many cities located in high Cl^−^ and high SO*_x_* environment in Asia, it is important to investigate the corrosion resistance of steels under this environment. The corrosivity and corrosion map was already identified in ISO 9223, where the exposure test using steel samples is conducted [1]. Besides, the adjustment of the classification system has been presented in progress based on ISO 9223 [2]. However, the characterization of the rust on steels at each site has not been conducted sufficiently.

Weathering steels are advantageous for reducing the maintenance cost of bridges and other infrastructure structures [3]. In addition, with the conventional weathering steel (SMA: 0.6% Cr-0.4% Cu-Fe), Ni bearing weathering steels have been proposed for applications in coastal environments [4]. Thus, there is the possibility for Ni bearing steel to also show high corrosion resistance in high SO*_x_* condition. However, there is few data concerning the corrosion performance of SMA and Ni bearing steel in high Cl^−^ and high SO*_x_* environment.

While there has been extensive analysis of rust on steels in mild environments [5,6,7,8,9,10,11,12], numerous questions remain regarding the basic mechanism of rust formation [4] and the effects of alloying elements [13,14,15] in severe environments. Indeed, several symposia have been held on the atmospheric corrosion of low alloy steels [15,16,17,18,19,20,21]. However, there have been no reports yet on the detail structure of the rust on low alloy steels [22,23,24] in high Cl^−^ and high SO*_x_* environment.

In this study, the rust formation on low alloy steels was investigated through the use of an actual exposure test in high Cl^−^ and high SO*_x_* environment. In particular, the nano structure of the rust on low alloy steel was examined by Raman spectroscopy and TEM (Transmission Electron Microscopy). Finally, the relationship between the formation of the rust and corrosion behavior was examined for low alloy steels exposed in high Cl^−^ and high SO*_x_* environment.

## 2. Materials and Methods

### 2.1. Test Samples and Exposure Corrosion Test

The low alloy steel was rough rolled at 1553 K, and then rolled at 1327 K to produce a 5 mm thick plate. The chemical composition (mass %) of low alloy steels was shown in Table 1. Here, KA1 and KA2 are 1% and 3% Ni steel, respectively; SMA is a conventional weathering steel (SMA: 0.6% Cr-0.4% Cu-Fe); and Carbon steel (SM) is for comparison.

The exposure test was conducted for three years at the exposure test site on Hainan Island in China. The corrosivity of Hainan area is C5 by ISO standards, which shows very high corrosivity. The test samples were exposed with a slope of 45 degree against the horizontal line.

**Figure 1:**
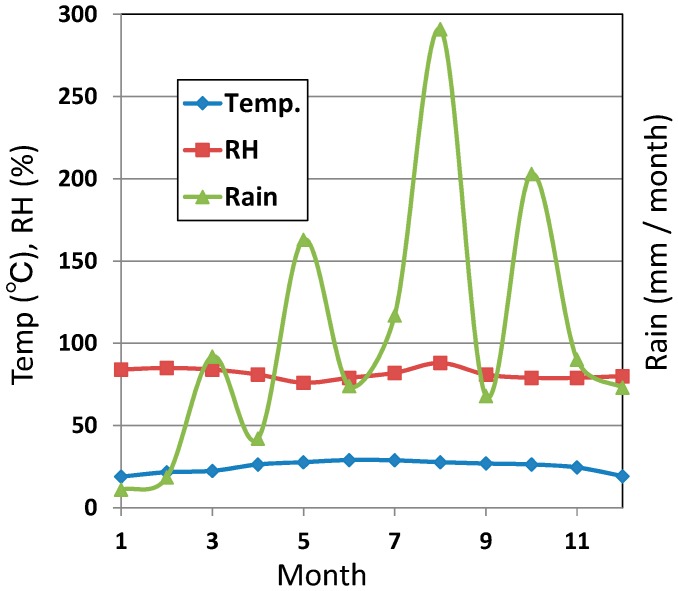
Climate dates of temperature, relative humidity (RH) and amount of rain in a year at the exposure site.

The annual climate data (temperature, RH (relative humidity), amount of rain) for the test site are shown in Figure 1. The average temperature is 19–29 °C, RH is almost 80%, and the amount of rain is high in summer, which is identified as a high humidity climate in the subtropical zone. 

**Figure 2:**
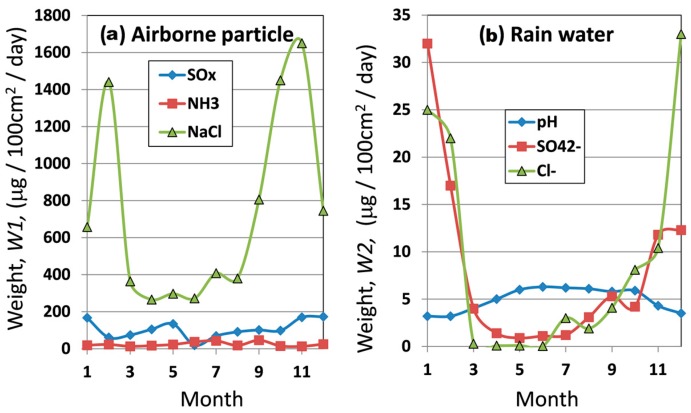
Corrosion factors of: (**a**) airborne particles; and (**b**) rainwater in a year at the exposure site.

Figure 2 shows corrosion factors of: (a) airborne particles; and (b) rain water in a year at the exposure site. The airborne particles are estimated as a weight in 100 cm^2^ in a day, showing that Cl^−^ is very high and SO*_x_* is to some extent high. The concentrations of SO_4_^2−^ and Cl^−^ in rainwater are examined in the unit of mg/m^3^, showing that concentration of SO_4_^2−^ and Cl^−^ are very high in winter. Accordingly, pH in rainwater is low in winter. Thus, the environment at the test site is defined as high Cl^−^ and high SO*_x_* condition in the subtropical climate zone.

The airborne particles are measured by collection using 10 × 10 cm^2^ gauze every month. Thus, these values are different from the contents in the rain as airborne particles come from the sea with the wind, which is different from the condition in the rain. 

After the exposure test, the extent of corrosion was determined by the reduction in thickness of the steel plate after removing the rust. The rust was first taken from the steel using a steel stick. Then, the steels were exposed in a diammonium hydrogen citrate solution (300 g/L, 60 °C) up to the surface of steels.

### 2.2. Physical Analysis of Rust

Surface analysis of the rust was conducted after the exposure test. The cross section of the rust was measured by SEM (Scanning Electron Microscopy). The EDS (Electron Dispersing Spectroscopy) was applied to investigate the concentration of various elements in the rust. As for the rust of Ni steel, Fe, Ni and Si were measured. In the case of SMA, Fe, Cr and Cu were measured. In addition, micro Raman spectroscopy was carried out with a 532 nm laser beam and a slit width of 25 μm. The frequency region was 4000–200 cm^−1^ to detect Fe oxides. The inner and outer rust were examined and compared. The measured peak positions were identified by using those of standard chemicals of iron oxides.

Nanostructure observation of the rust was performed by TEM analysis. The rust was cut by FIB (focused ion beam) from the inner rust. EELS (Electron Energy Loss Spectroscopy) analysis was conducted in order to identify the chemical state of elements and the nano structure in inner rust. In the case of Ni steel, the chemical shift of Ni was examined by Ni-L peak using standard chemicals of Ni and NiO. In the case of SMA, the chemical shift of Cr was examined by Cr-L peak. Additionally, the chemical shift of Oxygen and Fe were measured by O-K and Fe-L peaks. Finally, the nano structures of the rust of low alloy steels were discussed.

## 3. Results and Discussion

### 3.1. Corrosion of Low Alloy Steels in High SO_x_ Environment

**Figure 3:**
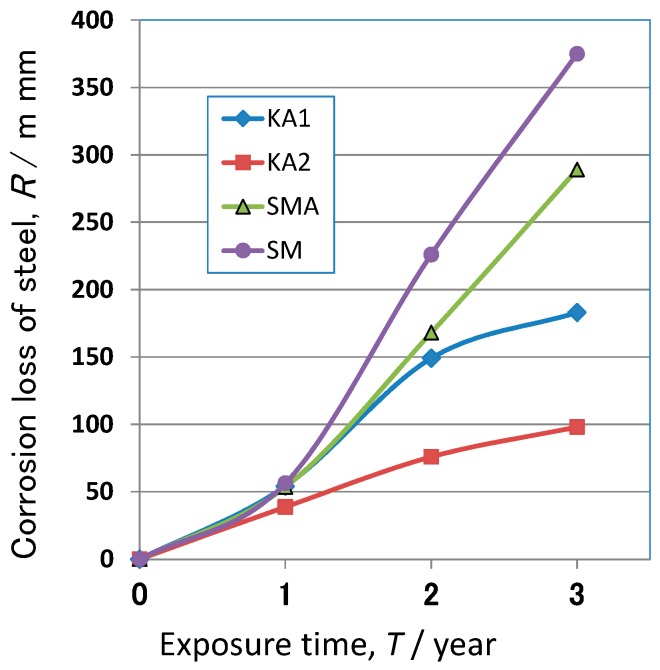
Exposure test results for low alloy steels at the test site for three years.

The corrosion resistance of the steels was estimated after the exposure test for three years at the test site. Figure 3 shows the exposure test results for KA1 (1% Ni), KA2 (3% Ni), SMA (0.6% Cr-0.4% Cu) and SM (carbon steel). The amount of corrosion of SM increases greatly with exposure time, and that of SMA is a little low compared to SM. On the other hand, the amount of corrosion of Ni bearing steel is less. Especially, KA2 (3% Ni) shows much less corrosion than other steels. Thus, Ni bearing steel exhibits excellent corrosion resistance in the exposure test as compared to SM. In other words, Ni bearing steel is recognized to be resistant to corrosion in high Cl^−^ and high SO*_x_* environment. 

### 3.2. Surface Analysis of the Rust Formed on Low Alloy Steels

**Figure 4:**
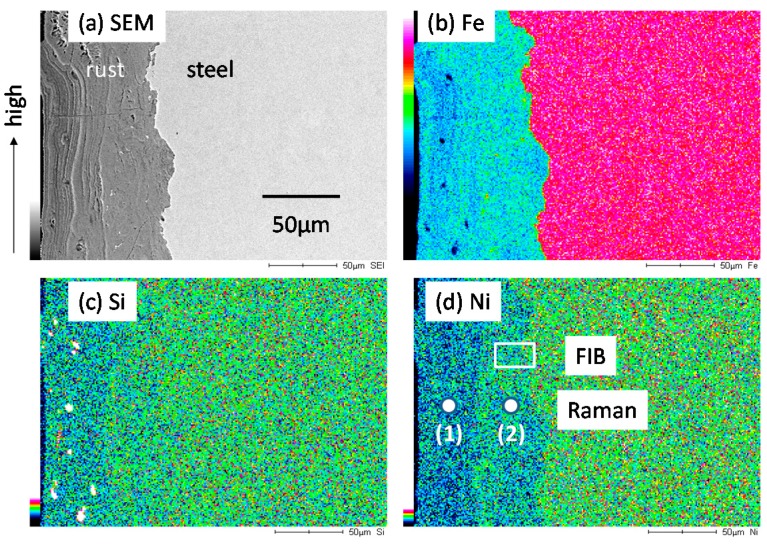
EDS (Electron Dispersing Spectroscopy) mapping of the rust formed on KA2 (3% Ni steel) after the exposure test for three years. (**a**) SEM (Scanning Electron Microscopy); (**b**) Fe; (**c**) Si; (**d**) Ni. FIB: focused ion beam.

To identify the corrosion preventing mechanism of rust on low alloy steels, the rust was estimated by surface analysis. Figure 4 shows SEM-EDS mapping for the rust on KA2 (3% Ni steel) after the exposure test for three years. Figure 4a is a cross section of SEM that indicates the location of the rust in the left side and the steel in the right one. Figure 4b–d is EDS mappings of the rust, showing the presence of Fe, Si and Ni. In Figure 4d, Ni is contained in the rust, and slightly enriched in inner rust. The experimental spots for Raman spectroscopy and area for FIB in TEM are shown in the figure. In Figure 4c, Si is contained in the rust; however, there is little enrichment. From above results, it is found that the rust on KA2 (3% Ni steel) contains Fe, Ni and Si.

**Figure 5:**
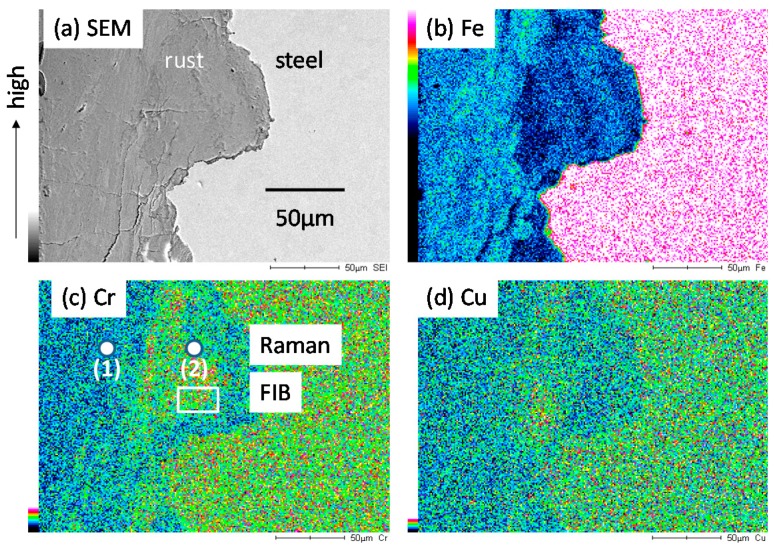
EDS mapping of the rust formed on SMA (0.6% Cr-0.4% Cu) after the exposure test for three years. (**a**) SEM; (**b**) Fe; (**c**) Cr; (**d**) Cu.

**Figure 6:**
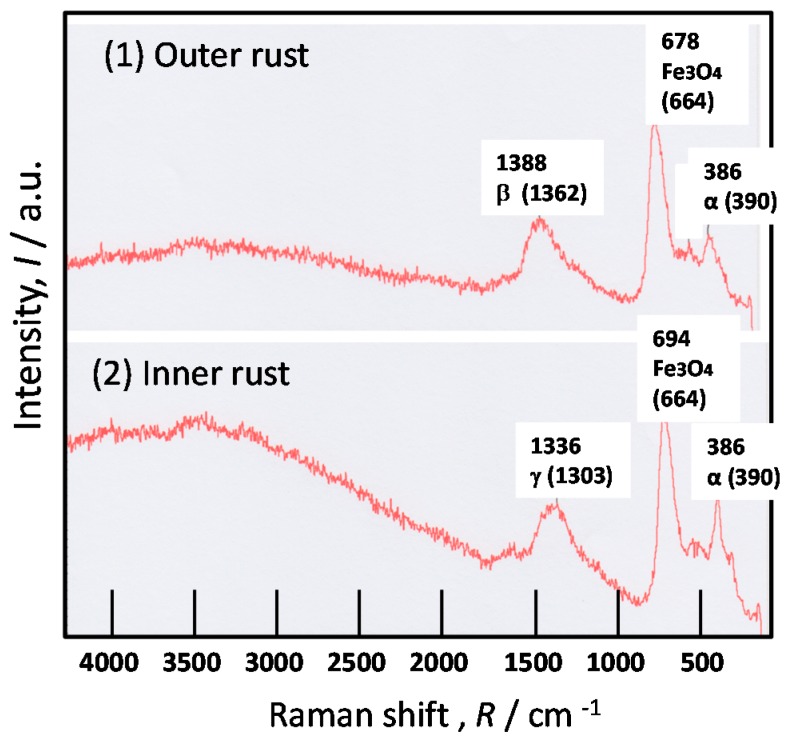
Raman results of the rust formed on KA2 (3% Ni steel) after the exposure test for three years. (**1**) Outer rust; (**2**) inner rust.

Figure 5 shows SEM-EDS mapping for the rust on SMA (0.6% Cr-0.4% Cu-Fe) after the exposure test for three years. Figure 5a is a cross section of SEM, and Figure 5b–d is EDS mappings of the rust, showing the presence of Fe, Cr and Cu. In Figure 6c, Cr is enriched in inner rust. The corrosion resistance of SMA is thought to relate the enrichment of Cr in inner rust. The experimental spots for Raman spectroscopy and area for FIB are shown in the figure. In Figure 5d, Cu is slightly enriched in inner rust. Thus, the rust on SMA contains Fe, Cr and Cu.

To identify the Fe oxides in inner and outer rust separately, the micro Raman spectra were measured for KA2 and SMA. Figure 6 shows Raman spectra at: (1) outer; and (2) inner rust on KA2 (3% Ni steel) after the exposure test for three years. At spot (2) in inner rust, the α-FeOOH (α), γ-FeOOH (γ) and Fe_3_O_4_ are detected. As the intensities of α-FeOOH and Fe_3_O_4_ are strong, the amounts of these oxides are thought high in inner rust. Besides, this Fe_3_O_4_ is thought to be the spinel Fe oxide, which is very fine particle. In general, α-FeOOH and Fe_3_O_4_ are thought to make the protective rust in inner rust. Thus, the inner rust of Ni bearing steel is thought to be composed of nano-size α-FeOOH and the spinel oxide, which increases the corrosion resistance of the rust. Similarly, at Spot (1) in outer rust, the α-FeOOH (α), β-FeOOH (β) and Fe_3_O_4_ are observed. β-FeOOH is thought made by the Chloride particles from the sea [25,26,27]. Although β-FeOOH decreases the corrosion resistance of steel, the protective inner rust can protect the steel. Thus, Ni bearing steel thought to have the corrosion resistant rust which is mainly composed of nano-size α-FeOOH and the spinel oxide in inner rust. 

**Figure 7:**
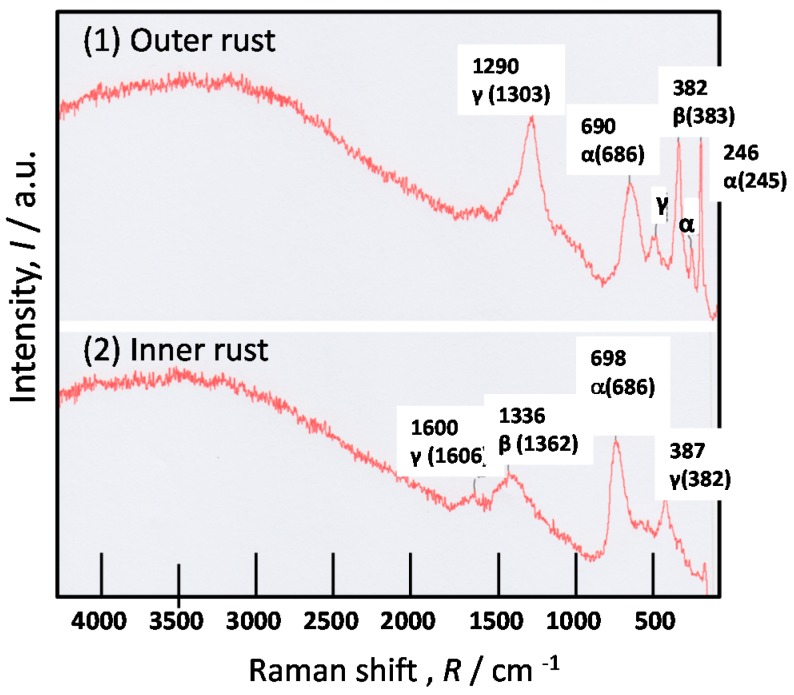
Raman results of the rust formed on SMA after the exposure test for three years. (**1**) Outer rust; (**2**) inner rust.

Figure 7 shows Raman spectra at spot (1) and (2) for the rust on SMA (0.6% Cr-0.4% Cu) after the exposure test three years. In the Raman results, the measured peak positions and those of standard chemicals are shown using values of Raman shift. The Raman peak positions for the standard chemicals of iron oxides have been measured in my laboratory. The peak positions between results and chemicals are well fit. The peaks of β- and γ-FeOOH around 1300–1360 are very close. However, they can be distinguished by the peak values β-FeOOH has the peak at 1362, and γ-FeOOH has one at 1303 cm^−1^.

At spot (2) in inner rust, α-FeOOH (α) is detected. Thus, the inner rust of SMA is thought to be composed of nano-size α-FeOOH, which increases the corrosion resistance of steel. However, β-FeOOH (β) and γ-FeOOH (γ) are recognized in inner rust, and there is no Fe_3_O_4_. Thus, the protection of the inner rust is considered less than Ni steel. At Spot (1) in outer rust, the strong peak of γ-FeOOH (γ) and β-FeOOH (β) are detected with peaks of α-FeOOH (α). As γ-FeOOH and β-FeOOH accelerates the corrosion of steels, the corrosion resistance of SMA is found to be less than Ni steel. Therefore, the corrosion resistance of the rust can be assumed using the estimation of Fe oxides measured by Raman spectra.

**Figure 8:**
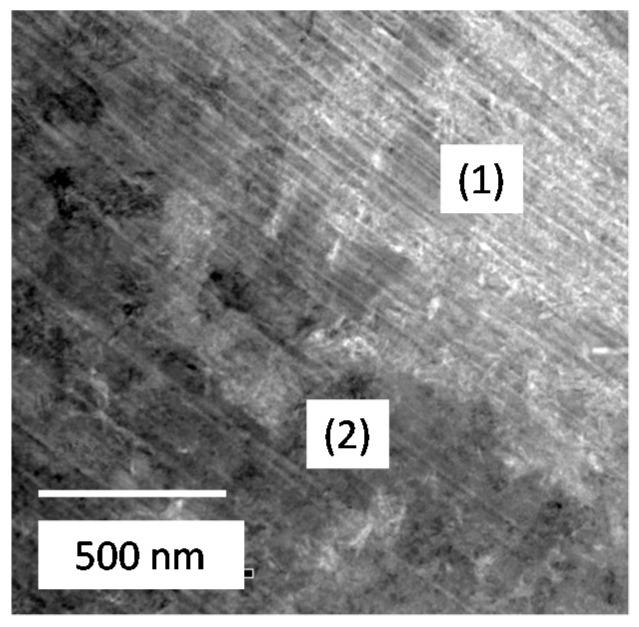
FIB-TEM (Transmission Electron Microscopy) observation taken from the position in Figure 4d for the rust of KA2 (3% Ni steel).

FIB-TEM analysis was conducted on the rust of low alloy steels after exposure test for three years. The sample of KA2 (3% Ni steel) was cut from the rust very near to the steel using a FIB, as shown in Figure 4. The experimental positions (1) and (2) for TEM-EELS (Electron Energy Loss Spectroscopy) are indicated in Figure 8. Position 1 shows white and Position 2 is dark, which reflects the chemical state of element at each position. In the following, EELS measurement was conducted at Positions 1 and 2.

**Figure 9:**
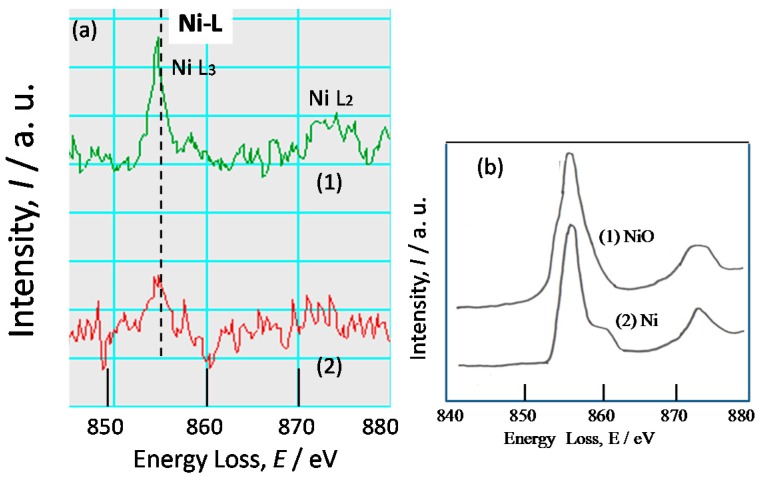
TEM-EELS (Electron Energy Loss Spectroscopy) spectra of Ni-L for the rust on: (**a**) KA2 (3% Ni steel); and (**b**) chemicals ((**1**) NiO and (**2**) Ni).

Figure 9a shows the TEM-EELS spectra of Ni-L in inner rust of KA2 (3% Ni steel) as shown at Positions 1 and 2 in Figure 8. Besides, Figure 9b shows Ni-L spectra of the standard chemicals of NiO and Ni used for comparison. Ni-L spectrum at Position 1 has a sharper peak than that at Position 2. Thus, Ni content is higher at Position 1 than Position 2. The spectra have a strong peak of *Ni-L*_3_ at 855 eV, which is just the same as those of NiO and Ni. Though the peak of *Ni-L*_2_ of KA2 at 874 eV is the same as those of NiO and Ni, the shape of the spectrum is similar to that of NiO. Thus, Ni is thought to exist as Ni(II) oxide state in inner rust of Ni bearing steel. 

**Figure 10:**
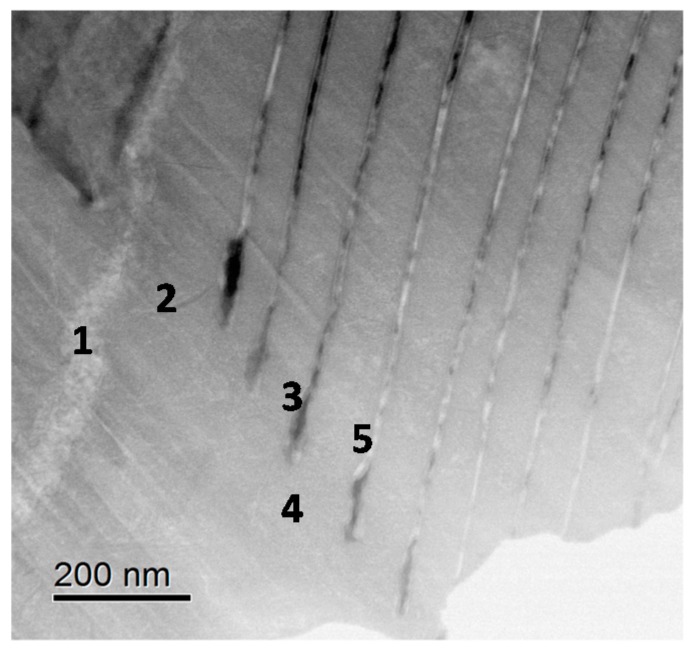
FIB-TEM observation taken from the position in Figure 5c for the rust of SMA.

FIB-TEM result for the rust of SMA (0.6% Cr-0.4% Cu-Fe) near the steel is indicated in Figure 10 corresponding to the position in Figure 5c. EELS measuring positions are shown in the figure at 1–5. As shown in the figure, a layer-by-layer structure is formed. Position 1 is in the narrow layer in white. Position 2 is in the wide layer. Position 3 is in the narrow layer in black. Position 4 is in the wide layer. Position 5 is in the narrow layer in white. They are thought to have each style and color reflecting the chemical state of element in the rust.

**Figure 11:**
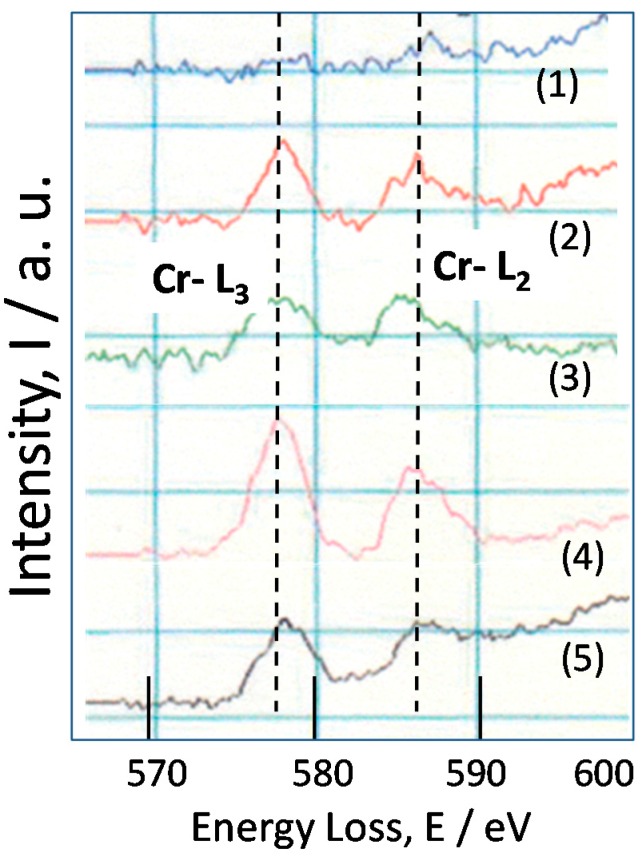
TEM-EELS spectra of Cr-L for the rust of SMA.

Figure 11 shows the EELS spectra of Cr-L at positions corresponding to those in Figure 10. The EELS spectra of Cr-L have peaks of Cr-L_3_ at 578 and Cr-L_2_ at 587 eV. In more detail, the peak of spectrum 3 is shifted, showing that the valence of Cr is different. In general, Cr-L_3_ and Cr-L_2_ of Cr(II)O are sifted to lower energy region as compared to those of Cr(III)O. From the previous paper [28], Cr(III)O has the peak of Cr-L_3_ at 580 and Cr-L_2_ at 589 eV, which are higher than the test results. Thus, Cr in Figure 11 is thought to contain Cr(III) and Cr(II) oxide state in the rust. There is no peak of Cr-L at Position 1, showing that the content of Cr is very less at this position. Therefore, the chemical statement of Cr in inner rust can be estimated by EELS measurement.

**Figure 12:**
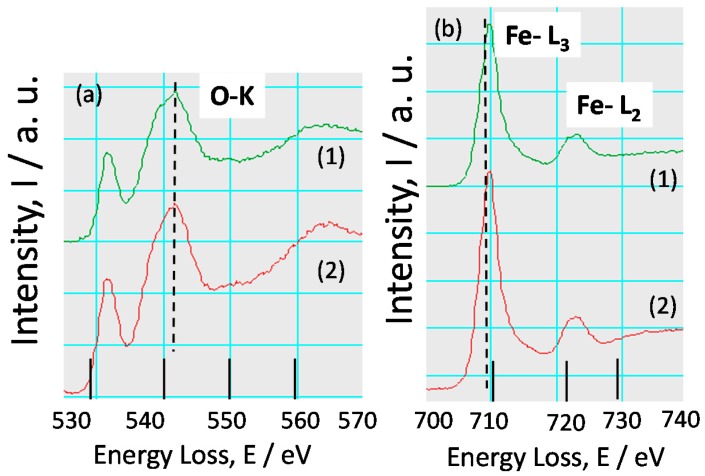
TEM-EELS spectra of: (**a**) O-K; and (**b**) Fe-L for the rust of KA2 (3% Ni steel).

In order to investigate the chemical state of Fe oxide in the rust, EELS spectra of O-K and Fe-L were observed as followings. TEM-EELS spectra of O-K (Figure 12a) and Fe-L (Figure 12b) for the rust of KA2 (3% Ni steel) at Positions 1 and 2 in Figure 8 are indicated in Figure 12. Both spectra of O-K have peaks at 532 and 542 eV, showing the oxidized state. In more detail, the shape of each spectrum differs a little from each other, demonstrating the concentration of Ni at each position is different. 

Figure 12b shows the EELS spectra of Fe-L at positions corresponding to those in Figure 8. Both spectra have peaks of Fe-L_3_ and Fe-L_2_, indicating the presence of Fe in the rust. In more detail, both spectra have peaks of Fe-L_3_ at 710 and Fe-L_2_ at 724 eV. The EELS spectra of Fe oxides including Fe(II)O and Fe(III)O has been reported in the previous papers [29]. Besides, the peaks of Fe-L_3_ and Fe-L_2_ are shifted to higher energy region in the case of Fe(III)O than Fe(II)O. The shapes of the spectra obtained here show the peak similar to that of Fe(III)O. Thus, Fe is thought to exist in inner rust of low alloy steel mainly as Fe(III) oxide state. 

**Figure 13:**
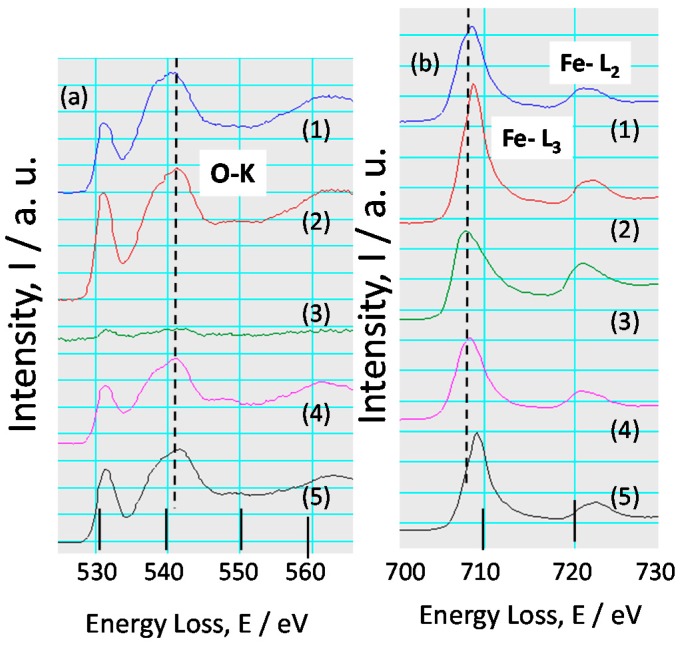
TEM-EELS spectra of: (**a**) O-K; and (**b**) Fe-L for the rust of SMA.

Figure 13 indicates TEM-EELS spectra of: (a) O-K; and (b) Fe-L, for the rust of SMA at positions 1–5 in Figure 10. Except spectrum 3, most spectra of O-K (Figure 13a) have peaks at 532 and 542 eV, showing the oxidized state. In more detail, the shape of each spectrum differs a little from each other, reflecting the concentration of elements at each position.

EELS spectra of Fe-L are shown in Figure 13b corresponding to those at positions in Figure 10. All spectra have peaks of Fe-L_3_ and Fe-L_2_, indicating the presence of Fe in the rust. In more detail, spectra 2, 4, and 5 have peaks of Fe-L_3_ at 709 and Fe-L_2_ at 723 eV. However, spectra 1 and 3 have positions of Fe-L_3_ at 708 and Fe-L_2_ at 722. Thus, the peak positions for spectra 1 and 3 are shifted to lower energy direction, which indicates that Fe(II)O is contained. The shape of the spectra obtained here are mainly similar to that of Fe(III)O, and a few are that of Fe(II)O in spectra 1 and 3. Thus, Fe is thought to exist in inner rust of low alloy steel as mainly Fe(III) and little Fe(II) oxide state. Raman results detected α-FeOOH in inner rust in Figure 7. As α-FeOOH contains only Fe(III)O, the higher content of α-FeOOH inner rust increases the presence of Fe(III)O. Thus, Fe is thought to exist in inner rust of low alloy steel as mainly Fe(III) and little Fe(II) oxide state. 

### 3.3. Corrosion Resistance Mechanism of Low Alloy Steels in High Cl^−^ and High SO_x_ Environment

The climate of the test site indicates high temperature and high RH (relative humidity), showing that of the sub tropical zone. Although the amount of airborne particles of NH_3_ is low, that of NaCl is very high. Besides, the amount of airborne particles of SO*_x_* is more than 100 mg/100 cm^2^/day. In rainwater, Cl^−^ and SO_4_^2−^ ions are high in winter season. The rainwater pH is low (4–6) in winter, showing that the corrosion of steels is increased by the acidic rain. Thus, the reason for high corrosion of carbon steel (SM) is thought to be caused by high Cl^−^ and high SO*_x_* in the environment. Moreover, RH at the exposure site is very high throughout one year, which promotes the increase of the corrosion of SM. Thus, the corrosion factors at the exposure test site are thought to be high Cl^−^, high SO*_x_* and high RH, which makes a very severe corrosion condition.

Ni bearing steel showed much higher corrosion resistance than SM in the high Cl^−^ and high SO*_x_* environment. The primary reason for this fact is likely to be the protective rust formed on Ni bearing steel during the exposure test. Thus, the high corrosion resistance of Ni steel is maintained in the Cl^−^ and SO*_x_* rich environment. By Raman spectroscopy, the rust of KA2 (Ni steel) was found to be mainly composed of nano-size α-FeOOH and spinel oxides. Even in outer rust of KA2, nano-size α-FeOOH and the spinel oxides were the primary components. Thus, Ni bearing steel had protective rust in this severe environment. On the other hand, in inner rust of SMA, β- and γ-FeOOH was detected, showing that corrosion resistance was not so high as compared to KA2. Provably, as the structure of inner rust of SMA is a little porous structure, chloride ions penetrates into the rust, and makes β- and γ-FeOOH. However, as SMA has much higher corrosion resistance than SM in the exposure test result in Figure 3, the protect ability of inner rust of SMA is thought effective using nano-size α-FeOOH.

TEM-EELS measurements were conducted to identify the chemical state of elements in inner rust of low alloy steels. The EELS spectra of Ni show that Ni exists as Ni(II) oxide state in inner rust of Ni bearing steel. In addition, SEM EDS indicated that Ni exists throughout the rust, and there is no localized enrichment. Ni likely exists as Ni(II) oxide state in all of the rust.

The EELS spectra of Cr show that Cr exists as mainly Cr(III) and a little Cr(II) oxide state in inner rust of SMA. Cr likely exists as Cr(III) oxide state in the α-FeOOH. The EELS spectra of Fe indicated the presence of Fe(III)O, suggesting that Fe exists as mainly Fe(III) and a little Fe(II) oxide state in inner rust. From Raman results, α-FeOOH is detected in inner rust which implies that Fe(III) oxide exists mainly in α-FeOOH in inner rust. 

Finally, it was demonstrated that the rust layer was composed of nano-size complex iron oxides containing Ni or Cr, which could prevent the corrosion of steel from Cl^−^ and SO*_x_*. Thus, these low alloy steels could form protective rusts against the corrosion in high Cl^−^ and high SO*_x_* environments.

## 4. Conclusions

Exposure tests were performed on low alloy steels in high Cl^−^ and high SO*_x_* environment, and the structure of the rust was analyzed by TEM and Raman spectroscopy.

1. In the exposure tests, Cl^−^ and SO*_x_* were dominant factors in the corrosion of steels, and a high relative humidity also had an effect. Besides, the corrosivity at the test site was C5 by ISO standards, which corresponds to the results of this paper.
2. The conventional weathering steel (SMA) showed lower corrosion weight loss as compared to the carbon steel (SM), and Ni bearing steel exhibited the lowest one.
3. Raman spectroscopy showed that the inner rusts on Ni bearing steel and SMA had α-FeOOH. Besides, Ni bearing steel had the spinel oxide in inner rust, which suppressed the corrosion.
4. TEM showed that the rust layer was composed of nano-size complex iron oxides containing Ni or Cr, which indicated that these low alloy steels formed protective rusts against the corrosion in high Cl^−^ and high SO*_x_* environments.

## Figures and Tables

**Table 1:** Chemical composition of the low alloy steels (mass %).

Number	Samples	C	Si	Ni	Cr	Cu
KA1	1% Ni Steel	0.1	0.2	1	-	-
KA2	3% Ni Steel	0.1	0.2	3	-	-
SMA	0.6% Cr-0.4% Cu	0.1	0.2	0.1	0.6	0.4
SM	Carbon Steel	0.1	0.2	-	-	-
